# Autologous chondrocyte implantation in the knee is effective in skeletally immature patients: a systematic review

**DOI:** 10.1007/s00167-022-07212-y

**Published:** 2022-11-03

**Authors:** Filippo Migliorini, Joerg Eschweiler, Julia Prinz, Christian David Weber, Ulf Krister Hofmann, Frank Hildebrand, Nicola Maffulli

**Affiliations:** 1 Department of Orthopaedic, Trauma, and Reconstructive Surgery, RWTH University Hospital, 52074 Aachen, Germany; 2 Department of Ophthalmology, RWTH University Hospital, 52074 Aachen, Germany; 3 Department of Medicine, Surgery and Dentistry, University of Salerno, 84081 Baronissi, SA Italy; 4 School of Pharmacy and Bioengineering, Faculty of Medicine, Keele University, ST4 7QB Stoke On Trent, England; 5 Barts and the London School of Medicine and Dentistry, Centre for Sports and Exercise Medicine, Mile End Hospital, Queen Mary University of London,, E1 4DG London, England; 6 Department of Orthopedics and Trauma Surgery, Eifelklinik St. Brigida, 52152 Simmerath, Germany

**Keywords:** Autologous chondrocyte implantation, ACI, Young, Pediatric, Skeletally immature

## Abstract

**Purpose:**

This systematic review evaluated the efficacy and safety of autologous chondrocyte implantation (ACI) for chondral defects of the knee in skeletally immature patients. Current available data from patients reported outcome measures (PROMs) and complications were collected, analyzed, and discussed.

**Methods:**

This systematic review was conducted according to the PRISMA guidelines. The following databases were accessed in May 2022: PubMed, Google scholar, Embase, and Scopus. All the clinical studies investigating the efficacy of ACI to manage chondral defects of the knee in skeletally immature patients were accessed. Articles treating patients with surgical procedures other than ACI were not eligible, nor were studies with a follow-up shorter than 12 months.

**Results:**

Data from 9 studies (251 procedures) were collected. 32% (80 of 251) of patients were females. The mean length of follow-up was 44.2 ± 29.4 (range, 12–115) months. The mean age of the patients was 16.4 ± 0.7 (range, 15–17) years. The Knee injury and Osteoarthritis Outcome Score (KOOS) and International Knee Document Committee (IKDC) increased of + 41.9/100 (*P* = 0.003) and + 33.2/100 (*P* =  < 0.0001) points, respectively. The Lysholm Knee Score improved of + 20.6/100 (*P* = 0.02) points. The Visual Analogue Scale (VAS) for pain reduced of − 3.6/10 (*P* = 0.004) points. The Tegner scale did not show any statistically significant improvement from baseline to follow-up (*P* = n.s.). The rate of graft hypertrophy was 12.5% (5 of 40 patients), and the rate of failure 5.6% (8 of 142 patients).

**Conclusion:**

ACI for chondral defects of the knee is effective to improve PROMs in skeletally immature patients. The safety profile of ACI still remains controversial.

**Level of evidence:**

III.

## Introduction

Chondral defects of the knee in skeletally immature patients are common and can occur following acute trauma, such as ankle sprain or patellar dislocation [42, 55]. Up to 10% of adolescents and young adults with knee hemarthrosis following acute trauma present a focal chondral damage [2]. Osteochondritis dissecans (OCD) is an idiopathic necrosis in a localized weight-bearing area of epiphyseal cartilage or subchondral bone and is the most common non-traumatic cause of focal chondral defect in skeletally immature patients [9, 18, 28, 58].

The management of chondral defects in young patients is challenging, with limited evidence and unpredictable outcomes. Regardless of its pathogenesis, autologous chondrocyte implantation (ACI) has been widely performed for chondral defects of the knee [17, 38]. ACI is a two-step surgical procedure. Chondrocytes are harvested from a non-weight-bearing zone of the knee in a fully arthroscopic fashion, and expanded externally in a laboratory [34, 41]. In a second surgical session, the chondral defect is debrided, and the expanded chondrocytes are implanted in the defect. A periosteal flap (I generation, pACI) or a collagen membrane (II generation, cACI) is used to retain the expanded chondrocytes into the defect [31, 38]. Matrix-induced ACI (mACI) represents the III generation of procedures: the harvested autologous chondrocytes are seeded and expanded directly on a resorbable membrane, which is subsequently implanted over the defect (mACI) [5, 33, 41, 47]. Current evidence on ACI in skeletally immature patients arises from a limited number of clinical studies, and, to the best of our knowledge, no previous systematic review has been conducted. Therefore, this systematic review evaluated the efficacy and safety profile of ACI for chondral defects of the knee in skeletally immature patients. Current available data from patient-reported outcome measures (PROMs) and complications were collected, analyzed, and discussed. It was hypothesized that ACI for the management of chondral defects of the knee is effective and safe in skeletally immature patients.

## Materials and methods

### Search strategy

This systematic review was conducted according to the Preferred Reporting Items for Systematic Reviews and Meta-Analyses: the PRISMA guidelines [46]. The PICOT algorithm was preliminary pointed out:

- P (Population): adolescents;
- I (Intervention): chondral repair surgical procedure;
- C (Comparison): improvement from baseline to last follow-up;
- O (Outcomes): PROMs and complications;
- T (Timing): minimum 12 month follow-up.

### Data source and extraction

Two independent authors (**;**) performed the literature search. The following databases were accessed in May 2022: PubMed, Google scholar, Embase, and Scopus. The following keywords were used: knee, chondral, cartilage, hyaline, defects, damage, injury, pain, symptoms, surgery, autologous chondrocyte implantation, ACI, management, strategy, therapy, treatment, regeneration, visual analogue scale, reoperation, revision, failure, hypertrophy, and delamination in combination using the Boolean operator (AND/OR). No time constrains were used for the search. The same authors independently performed the initial screening of the resulting titles. If the title and abstract matched the topic, the article full text was accessed. The bibliography of the full-text articles was also screened by hands. Disagreements were debated and mutual solved by consensus.

### Eligibility criteria

All the clinical studies investigating the efficacy and safety of ACI to manage chondral defects of the knee in skeletally immature patients were accessed. Articles in English, German, Italian, French, and Spanish were eligible. Levels I–IV of evidence, according to Oxford Centre of Evidence-Based Medicine [20], were considered. Only studies published in peer-reviewed journals were considered. Reviews and meta-analyses, editorials, commentaries, abstracts, and posters were not considered. Articles reporting patients treated with surgical procedures other than ACI were not eligible, nor were studies with follow-up shorter than 12 months. Studies which involved patients with closed growth plates were not considered. Only articles reporting quantitative data on the outcomes of interest were considered in the present systematic review.

### Outcomes of interest

Two independent authors (F.M. & J. E.) examined articles resulting from the literature search. Study generalities were collected: author, year, journal, and type of study. Patient baseline data were also retrieved: number of samples, mean BMI and age, mean duration of the symptoms, mean length of the follow-up, percentage of female, and mean size of the defect (cm^2^). Data with regards to the following PROMs were collected: Knee injury and Osteoarthritis Outcome Score (KOOS) [57], Visual Analogue Scale (VAS), Tegner Activity Scale [8], Lysholm Knee Score [11], and the International Knee Document Committee (IKDC) [22]. Data concerning the rates of failure and graft hypertrophy were also collected.

### Methodology quality assessment

The risk of bias was assessed by two authors independently (F.M. & J.E.). The methodological quality assessment was made through “Risk of Bias in Non-randomized Studies of Interventions” (ROBINS-I) scoring system [48]. The following biases were evaluated: confounding, selection of participants, classification of interventions, deviation from intended interventions, missing data, measurement of outcomes, and selection of the reported results.

### Statistical analysis

The statistical analyses were conducted by the main author (F.M.) using the software IBM SPSS version 25. For descriptive statistics, the arithmetic mean and standard deviation were used. For continuous variable, the mean difference (MD) effect measure was adopted. The standard error of the mean (SEM) was also evaluated. The confidence interval (CI) was set at 95%. The *t* test was evaluated, with values of *P* < 0.05 considered statistically significant.

## Results

### Search result

**Fig. 1:**
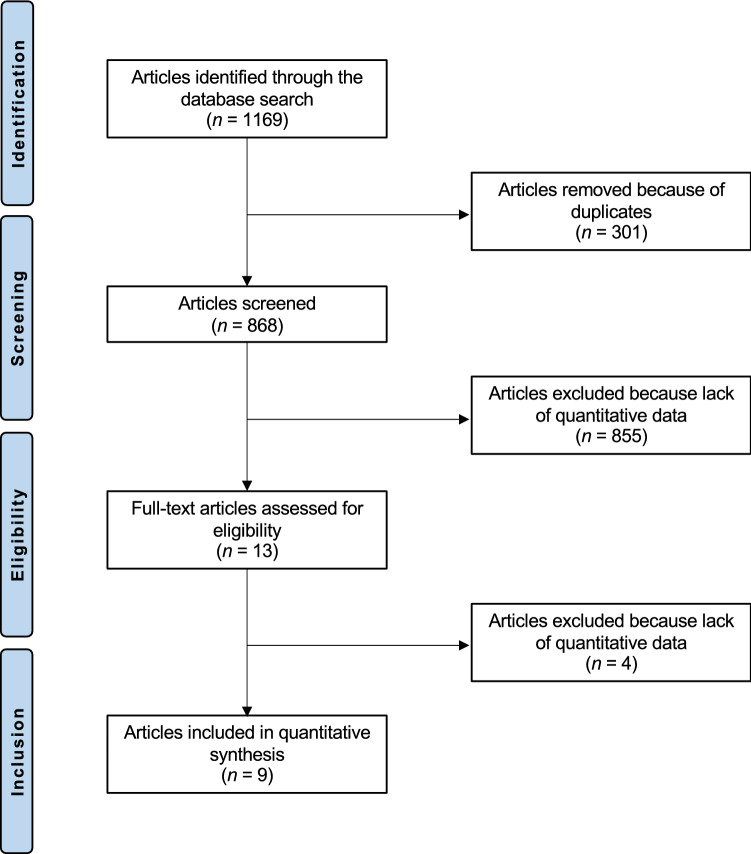
Flowchart of the literature search

The literature search produced 1169 articles. Of them, 301 were excluded as they were duplicates. Additionally, 855 studies were excluded with reason: reported data on other procedures rather than ACI (*N* = 18), reported data on adults with closed growing plates (*N* = 801), not compatible design or journal (*N* = 31), short follow-up (*N* = 2), and language limitations (*N* = 3). A further four studies were excluded as they did not report data under the outcomes of interests. Finally, nine studies were included in the present investigation: three prospective and six retrospective clinical investigations (Fig. 1).

### Methodological quality assessment

Given the retrospective nature and the lack of control group of most included studies, pre-intervention bias reported a moderate risk of bias. The overall bias at intervention was low. The intervention protocol was well defined in most studies, and no significant deviation from the intervention protocol was detected. Data were satisfactory reported in most included studies, and the clinical outcome assessment was comparable among the intervention groups. The reported results corresponded to the planned protocol in all included studies. Despite some limitations, all studies selected have an acceptable quality assessment (Table 1).

**Table 1:** Methodological quality assessment

Author, year	Pre-intervention risk of bias		At-intervention risk of bias	Post-intervention risk of bias				Overall
	Confounding	Selection	Classification of interventions	Deviations from intended interventions	Missing data	Measurement of outcomes	Reporting	
Cvetanovich et al. 2016 [12]	Moderate	Moderate	Low	Low	Low	Low	Low	Low
Dai et al. 2012 [13]	Moderate	Moderate	Low	Low	Serious	Low	Moderate	Moderate
Hoburg et al. 2019 [19]	Low	Moderate	Low	Low	Low	Low	Low	Low
Macmull et al. 2011[29]	Low	Moderate	Low	Low	Low	Low	Low	Low
Mithofer et al. 2005 [45]	Low	Moderate	Low	Low	Low	Low	Low	Low
Micheli et al. 2006 [30]	Moderate	Moderate	Low	Low	Low	Low	Low	Moderate
Niethammer et al. 2017 [51]	Low	Moderate	Low	Low	Low	Low	Low	Low
Ogura et al. 2017 [52]	Low	Moderate	Low	Low	Low	Low	Low	Low
Teo et al. 2013 [59]	Moderate	Serious	Low	Low	Low	Low	Low	Moderate

### Patient demographics

Data from 251 procedures were collected. 32% (80 of 251) of patients were females. The mean length of follow-up was 44.2 ± 29.4 (range, 12–115) months. The mean age of the patients was 16.4 ± 0.7 (range, 15–17) years. The mean BMI was 23.0 ± 1.2 kg/m^2^. Generalities of the included studies and patient demographic are shown in Table 2.

**Table 2:** Generalities of the included studies and patient baseline of the included studies

Author, year	Journal	Study Design	Treatment	Patients	Follow-up (months)	Female (%)	Mean age	Mean defect size (cm2)
Cvetanovich et al. 2016 [12]	Am J Sports Med	Prospective	pACI, cACI	37	24	22	17	4
Dai et al. 2012 [13]	Chin Med J	Retrospective	mACI	7	12	29	17	7.1
Hoburg et al. 2019 [19]	Orthop J Sports Med	Prospective	mACI	29	63	48	16	4.6
Macmull et al. 2011 [29]	Am J Sports Med	Prospective	pACI, cACI (24) mACI (7)	24	66	29	16	
Mithofer et al. 2005 [45]	Am J Sports Med	Retrospective	pACI	20	47	25	16	6.4
Micheli et al. 2006 [30]	Clin J Sport Med	Retrospective	pACI	37	51	40	15	5.3
Niethammer et al. 2017 [51]	Int Orthop	Retrospective	mACI	40	36	40	16	5.3
Ogura et al. 2017 [52]	Am J Sports Med	Retrospective	cACI	27	115	52	16	6.2
Teo et al. 2013 [59]	Clin Orthop Rel Res	Retrospective	pACI	20	24	20	17	

### Outcomes of interest

KOOS and IKCD increased of + 41.9/100 (*P* = 0.003) and + 33.2/100 (*P* =  < 0.0001) points, respectively. The Lysholm improved by + 20.6/100 (*P* = 0.02) points. The VAS reduced by -3.6/10 (*P* = 0.004) points. The Tegner scale did not show any statistically significant improvement from baseline to follow-up (n.s.). The rate of graft hypertrophy was 12.5% (5 of 40 patients), and the rate of failure 5.6% (8 of 142 patients). Results of PROMs are shown in greater detail in Table 3.

**Table 3:** Result of PROMs

Endpoint	Pre-operative	Last follow-up	MD	95% CI	SEM	*P*
KOOS (0–100)	23.9 ± 3.1	65.8 ± 17.2	41.9	38.45–45.34	1.75	0.003
VAS (0–10)	5.7 ± 0.8	2.1 ± 1.0	− 3.6	− 3.85 to − 3.34	0.13	0.004
Tegner (0–10)	3.2 ± 0.9	5.9 ± 2.7	2.8	2.13–3.26	0.29	n.s.
Lysholm (0–100)	57 .9 ± 9.9	50.1 ± 13.6	20.6	17.50–23.69	1.57	0.02
IKDC (0–100)	39.5 ± 6.6	72.6 ± 7.7	33.2	31.00–34.99	1.01	< 0.0001

*MD* mean difference, *CI* confidence interval, *SEM* standard error of the mean, *n.s.* not significant

## Discussion

According to the main findings of the present systematic review, ACI for chondral defects of the knee is effective to improve PROMs in skeletally immature patients. The mean improvement at last follow-up of the KOOS, IKDC, Lysholm, and VAS overcame their minimally clinically important difference (MCID) [1, 24, 49]. The Tegner scale did not show any statistically significant improvement from baseline to the last follow-up. The overall rate of complications was concerning: 12.5% of patients demonstrated graft hypertrophy and 5.6% of procedures failed. However, only a few authors reported data on the rate of complication. It has been hypothesized that some authors did not state clearly whether complication was found, thus producing an underestimation of the overall rate of complications. Many authors reported data on pACI, which is associated with a greater rate of graft hypertrophy than the other generations of ACI [4, 27, 60]. Moreover, the membrane was sutured in the first two generations of ACI (pACI and cACI) [33]. Although suturing allows a more stable fixation of the membrane, it produces partial-thickness lesions of the articular cartilage which may not heal and may enlarge with time, leading to pain, and premature osteoarthritis [21, 43]. Compared to the previous generations, mACI allows less invasive approaches (mini-arthrotomy or arthroscopy), avoids graft suture, and allows shorter surgical time [39].

At approximately 16 years, the growth plates of the femur start to fuse, and complete bone maturity is evidenced in 88% of subjects within 2 years, and 100% within 3 years [40]. The mean age of the patients included in the present study was 16 years; hence, most of the population had not yet reached bone maturity. In skeletally immature patients, conservative management may alleviate symptoms for a limited period, usually several in months [25].

Skeletally immature patients present higher bone marrow-derived stem cell concentration compared to adults, and regeneration can be stimulated by physiotherapy [3, 15, 54]. In addition, the open epiphyseal growth plates ensure a higher self-healing capability of the lesions (International Cartilage Repair Society, ICRS or Outerbridge grades I or II) [44]. In this respect, bone marrow stimulating procedures should be considered for skeletally immature patients. An isolated microfracture is a bone marrow stimulating technique widely used in adults with smaller defects [7, 56]. The blood clot of microfractures is unstable in bigger defects [14, 16]. To overcome these problems, in 2005, Autologous Matrix-Induced Chondrogenesis (AMIC) has been introduced [6]. In adult patients with focal chondral defects of the knee, AMIC showed promising results at midterm follow-up [35, 36]. AMIC has several advantages compared to ACI. ACI procedures are technically demanding, require two surgical sessions, and are burdened by donor site morbidity, higher costs, and long recovery times. AMIC is a bone marrow stimulating procedure which avoids cartilage harvest and external expansion, and is performed in a single surgical session [36, 37]. However, the current literature lacks studies evaluating bone marrow stimulating procedures in skeletally immature patients, and further studies are recommended.

The present study certainly has limitations. The retrospective nature of most of the included studies carries the risk of selection bias. Given the lack of quantitative data, it was not possible to conduct the statistical analyses including only prospective studies. Although the included studies were methodologically well conducted, most of them involved a limited sample size. The description of the diagnoses, eligibility criteria, and surgical procedures were overall satisfactory in most studies; however, information on rehabilitation protocols was seldom reported and often biased, and the timing of assessing outcome was often unclear. Between studies heterogeneities are evident. One study investigated surgical procedures on patients with chondral defects [12], others combined patients with chondral and osteochondral injuries [19, 29, 45, 59]. The impact on the surgical outcome of adding a subchondral bone procedure to ACI is not fully clarified. Subchondral bone impairment is always present in OCD defects, but not so common following traumatic chondral injuries. One article focused exclusively on patients with OCD [59], and many authors did not clearly state the aetiology of the defects. Whether the aetiology (OCD or trauma) influences the clinical outcome is unclear; given the limited available data, no further subgroup analyses were possible. The location of the defects was variable among the included studies: one study evaluated the patellofemoral joint [59], two the femoral condyles [30, 45], and six investigated mixed locations [12, 13, 19, 29, 51, 52]. Most authors performed the interventions in combination with other procedures [12, 13, 19, 45, 52], or did not clearly state whether the procedures were performed in isolation. A resorbable collagen I/III porcine-derived membrane was used by most authors [12, 13, 19, 29, 45, 52, 59]. One author [19] used 3-dimensional chondrospheres (Spherox), which adhere directly to the subchondral bone without the need of a membrane to stabilize the implant. Two authors combined data of patients who underwent pACI and cACI [12, 29], as they evolved their technique during the patient recruitment. Given the limited data available for inclusion, no further subgroup analyses were possible. The IKDC and the KOOS questionnaires evaluate pain, symptoms, and function in daily life and sport activities, and are widely employed to evaluate patients with chondral defects of the knee [7, 10, 32, 50]. Previous studies demonstrated their validity, reliability, and responsiveness in the adult population [11, 22, 57]. However, the validity of IKDC and KOOS in skeletally immature patients is controversial [23]. Therefore, the child friendly Pedi-IKDC [26] and KOOS-Child were developed and validated [53]. However, none of the included studies referred to the child friendly PROMs, and this represents a further bias. Therefore, these results must be considered within the limitations of the present investigation. Future investigations should validate these results in a clinical setting.

## Conclusion

ACI for chondral defects of the knee is effective to improve PROMs in skeletally immature patients. However, the safety profile of ACI still remains controversial, and needs to be clarified by further investigations.

## Data Availability

The data underlying this article are available in the article and in its online supplementary material.

## Abbreviations

ACI: Autologous chondrocyte implantation
PROMs: Patient-reported outcome measures
KOOS: Knee injury and osteoarthritis outcome score
IKDC: International knee document committee
VAS: Visual analogue scale
MD: Mean difference
SEM: Standard error of the mean
CI: Confidence interval
OCD: Osteochondritis dissecans
pACI: Periosteal flap ACI
cACI: Collagen membrane ACI
mACI: Matrix-induced ACI
n.s.: Not significant
